# *T*-shaped double-strip spoof surface plasmon polariton transmission lines and application to microwave resonators

**DOI:** 10.1038/s41598-022-11751-2

**Published:** 2022-05-09

**Authors:** Wonseok Choi, Jinho Jeong

**Affiliations:** Department of Electronic Engineering, Sogang University, Seoul, 04107 Korea

**Keywords:** Electrical and electronic engineering, Engineering

## Abstract

A microwave double-strip spoof surface plasmon polariton (DS SSPP) is proposed for high-speed interconnects and high-performance microwave circuits. Based on the dispersion analysis, a *T*-shaped double-strip structure is designed to provide strong surface- and slow-wave properties from very low to very high frequencies (~ 40 GHz). It allows the tight field confinement and greatly reduces the electromagnetic wave leakage. It exhibits broadband performance with reduced ripples in the insertion loss. It also shows more constant group delay and impedance than counterpart single-strip SSPP. The compact coaxial-to-microstrip-to-DS SSPP transition are designed using gradient grooves. The measurement shows that the DS SSPP lines can exhibit the lower coupling and lower insertion loss than the microstrip lines, so that the former is well-suited for the densely packed high-speed interconnects. The designed DS SSPP is utilized for high quality (*Q*)-factor microwave ring resonator. The measured unloaded *Q*-factor is 107.9 at the resonant frequency of 8.7 GHz, which is 1.3 times higher than the microstrip ring resonator. It is found to be caused by the reduction of the radiation loss, according to the loss analysis. The size is also reduced due to the short wavelength, occupying 56.8% of that of the microstrip ring resonator. Therefore, the proposed *T*-shaped DS SSPP can be also applied for high-performance miniaturized microwave circuits.

## Introduction

For high data-rate wireline transmissions, it is strongly required to design broadband and low-loss interconnects with a high-level integration under a compact area. Microstrip lines are the most popular transmission lines used for high-speed interconnects and microwave circuits as well, because they are compact, light-weight, and easy to fabricate^1^. They can be also easily integrated with active and passive devices. However, microstrip lines exhibit the increased insertion loss at high frequencies and the increased coupling or cross-talks in the densely packed interconnects, which limit the bandwidth of the interconnects, increase the interferences, and degrade the signal integrity^2^.

In order to meet the demand for high data-rate interconnects under a compact size, there have been researches to apply the surface plasmon polaritons (SPPs) for the high-speed interconnects^3^. The SPPs are kind of surface waves propagating along the interface between dielectric and conductor with negative permittivity at optical frequencies^4^. However, the SPP cannot exist at microwave frequencies at which the conductors are close to perfect electric conductor (PEC)^4^. Instead, SPP-like transmission lines have been proposed by using sub-wavelength periodic structures such as grooves and holes^2^, which are called spoof SPPs (SSPPs) or designer SPPs. A variety of the SSPP lines consisting of single metal strip on dielectric substrate, or single-strip SSPP (SS SSPP) lines, have been proposed at microwave frequencies^5–8^. The SS SSPP can propagate in transverse magnetic (TM) mode as like SPP at optical frequencies^4^. They also preserve the characteristic of surface waves so that the electromagnetic (EM) fields exponentially decay in the directions perpendicular to that of wave propagation. That is, the EM fields are strongly confined in the interface between metal strip and dielectric. It leads to the reduction of dielectric loss, compared to the microstrip line in which most of the EM fields are confined in the dielectric substrate with a high dielectric constant. The tight field confinement also results in the reduction of the coupling between closely placed lines, reducing the cross-talks and improving signal integrity^7^. The SS SSPP lines also exhibit slow-wave property, so that the wavelength is shorter than that of transverse electromagnetic (TEM) modes in the same medium^5–8^. Therefore, it is helpful in reducing the size of the microwave circuits.

However, the SS SSPP lines do not have a ground plane or line, so that it is difficult to be integrated with traditional TEM-mode transmission lines and active circuits. They also exhibit the impedance (Bloch impedance) much higher than the reference impedance of 50 Ω of the traditional systems, so that the large transition structures are needed for impedance transformation^5,9^. In addition, they show slow-wave characteristics only above a certain frequency (called intersection frequency), so that they are not well-suited for the broadband applications^3^.

In order to solve these problems of the SS SSPP lines, double-strip SSPP (DS SSPP) lines have been proposed, where the patterned ground line is added to form two-conductor transmission lines. Therefore, they can be easily integrated with active devices by using simple transition structures^3,9^. The analysis of two closely placed DS SSPP lines were performed to show the reduced coupling and suppressed interferences between the lines^3^. However, the reported DS SSPP lines above were designed at relatively low microwave frequencies (lower than 20 GHz) using simple structures such as comb- or bar-type unit cells. They also exhibit large fluctuations in the insertion loss especially at low frequencies (0.3–3.1 GHz) due to the mismatches by the transition between the microstrip and SSPP lines^9^. For higher speed data transmission and millimeter-wave applications, where the EM wave leakage and cross-talks can seriously degrade the signal integrity and microwave-circuit performance, the tighter field confinement is highly required by designing more sophisticated unit cells.

In this work, we propose a *T*-shaped DS SSPP which allows the tight coupling between adjacent cells to enhance slow-wave characteristics and to greatly reduce the EM wave leakage. It shows no intersection frequency, the reduced ripples in the insertion loss, and more constant group delay, so that it allows a broadband performance up to ~ 40 GHz. It also leads to the reduction of the wavelength compared to both microstrip and SS SSPP lines, which is beneficial for a miniaturization of microwave circuits. The proposed DS SSPP is utilized to design the microwave ring resonator with higher quality (*Q*)-factor than microstrip ring resonator, which is caused by the reduced radiation loss according to the loss analysis.

The *T*-shaped unit cell of DS SSPP is designed based on the dispersion analysis, exhibiting a strong slow-wave property with a cutoff frequency over 40 GHz. The performances of single straight line and coupled lines using the designed DS SSPP are compared with those of microstrip and SS SSPP lines in the simulations. The loss analysis is carried out to find the contributions of the conductor, dielectric, and radiation losses to the total loss. The transition structure is also designed using gradient grooves based on the dispersion analysis. Then, experimental results for single straight and coupled lines in microstrip and DS SSPP are presented and compared. The proposed DS SSPP line is applied to design the compact high-$Q$ microwave resonator of which the measured performance is compared with the conventional microstrip ring resonators.

## Results

### Design of double-strip spoof SPP transmission lines

**Figure 1:**
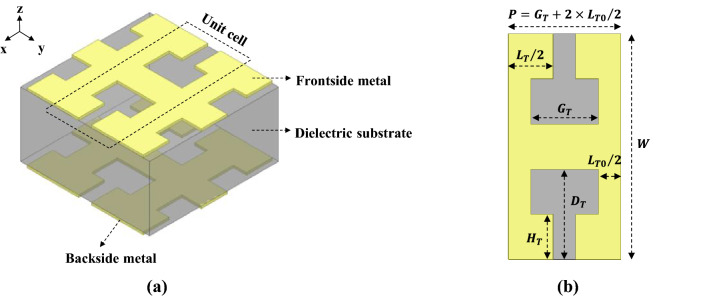
Unit cell of designed DS SSPP. (**a**) DS SSPP with *T*-shaped metal films on both sides of dielectric substrate. (**b**) Metal strip in which $D_{T}$ = 0.4 mm, $W$ = 1.0 mm, $G_{T}$ = 0.3 mm, $P$ = 0.5 mm, $H_{T}$ = 0.2 mm, $L_{T}$ = 0.4 mm, $L_{T0}$ = 0.2 mm.

Figure 1a shows the proposed unit cell of the DS SSPP transmission line in this work, consisting of the *T*-shaped metal strips printed on both sides of the dielectric substrate. The top and bottom metal strips have the same dimensions which are denoted in Fig. 1b. The unit-cell structure was designed to have SSPP characteristics with a high cut-off frequency, based on dispersion analysis using the commercial 3-dimensional EM structure simulator (HFSS by Ansys, Inc.). In this work, a high-TG FR-4 substrate was used in the design, which has a dielectric constant ($\varepsilon_{r}$) of 4.6, loss tangent ($\tan \delta$) of 0.007, and the thickness of 0.5 mm. The *T*-shaped metal strips are patterned in an 18 μm-thick copper layer.

**Figure 2:**
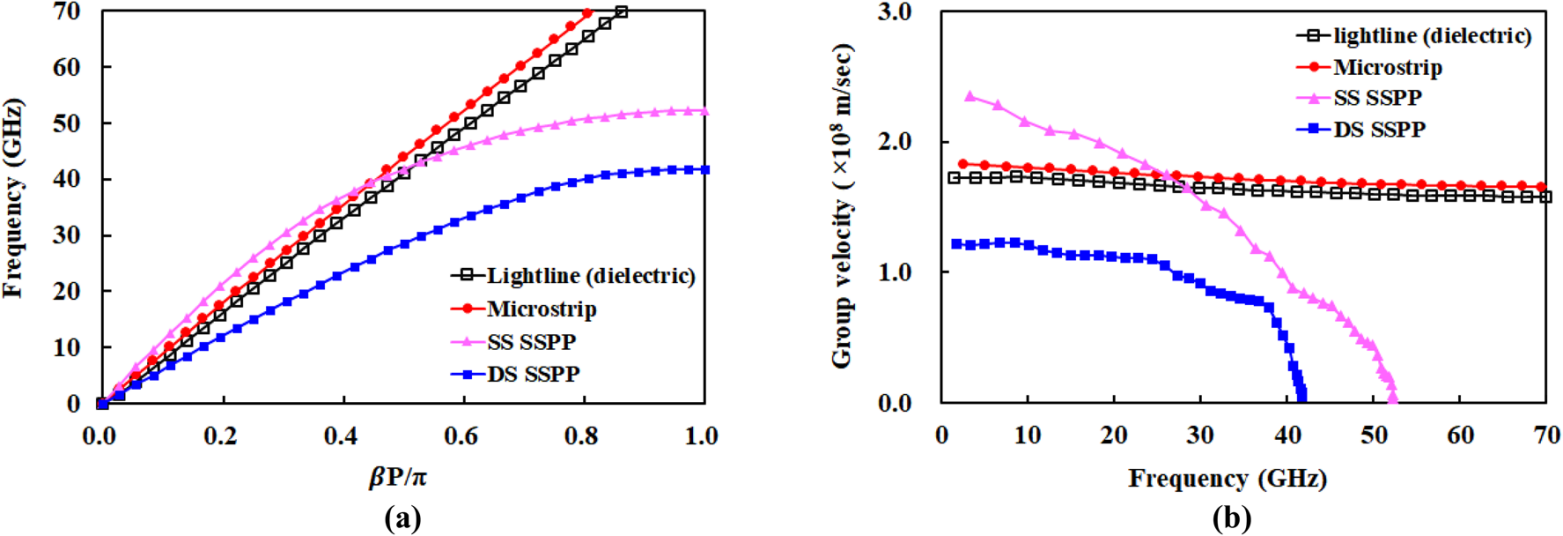
Dispersion curve and group velocity of lightline, microstrip, SS SSPP, and DS SSPP in the same dielectric substrate. (**a**) Dispersion curve. (**b**) Group velocity.

Figure 2a shows the simulated dispersion characteristics of the designed unit cell of DS SSPP, the plot of frequency versus $\beta P/\pi$, where $\beta$ and $P$ represent the wavenumber in the direction of $\pm y$-axis and the length of unit cell, respectively. The cut-off frequency at which $\beta P/\pi = 1$ is selected to be 47.9 GHz. For the comparison, the dispersion curves for various lines in the same substrate such as lightline, microstrip line, and the SS SSPP line are also included in this figure. The SS SSPP consists of a single metal strip on only one side of the substrate with the same dimensions as those of the DS SSPP. Note that the dispersion curves of the SS SSPP and the microstrip intersect at 38.6 GHz (intersection frequency). The SS SSPP line shows a larger $\beta$ than that of the microstrip line (quasi-TEM mode) only above the intersection frequency where the SS SSPP exhibits a slow wave characteristic with a high field confinement. Therefore, the SS SSPP line is not adequate for high-speed interconnects requiring a broadband performance from very low to very high frequencies.

On the contrary, the DS SSPP structure presents the SSPP mode from very low to cut-off frequencies without intersection frequency. Moreover, its curve is below those of the microstrip and SS SSPP over entire frequency range, implying the larger wavenumber and the enhanced slow-wave characteristics. It entails the shorter wavelength, allowing the miniaturization of the microwave circuits. According to Eq. (1), the larger wavenumber of the DS SSPP also results in the higher attenuation constant ($\alpha$) in the perpendicular direction of the wave propagation (for example, $z$-axis in Fig. 1a).

1 $$ \alpha = \sqrt {\beta^{2} - k_{0}^{2} \mu_{r} \varepsilon_{r} } , $$

where $k_{0}$ and $\mu_{r}$ are wavenumber in free space and relative permeability of the dielectric substrate, respectively. That is, the EM fields in the DS SSPP exponentially decay in air with a higher rate than the SS SSPP. Therefore, they are more tightly confined in the metal strips, forming a strong surface wave. It leads to the reduction of the radiation loss in the transmission lines and the cross-talks between densely packed interconnects.

For the high-speed signal transmission without distortion, it is required to maintain a constant phase velocity with respect to frequency. As shown in Fig. 2a, the dispersion curve of the DS SSPP is more linear than that of the SS SSPP, indicating more constant phase velocity. Figure 2b shows the group velocity ($v_{g} = d\omega /d\beta$) of the various transmission lines which are obtained from Fig. 2a. The TEM-mode transmission lines such as lightline and microstrip lines exhibit almost a constant group velocity. On the contrary, the SPP structures inherently exhibit variable group velocity with frequency. However, the DS SSPP presents the smaller variations in group velocity than the SS SSPP, implying that the former performs better than the latter for the high-speed data transmissions with less distortion.

**Figure 3:**
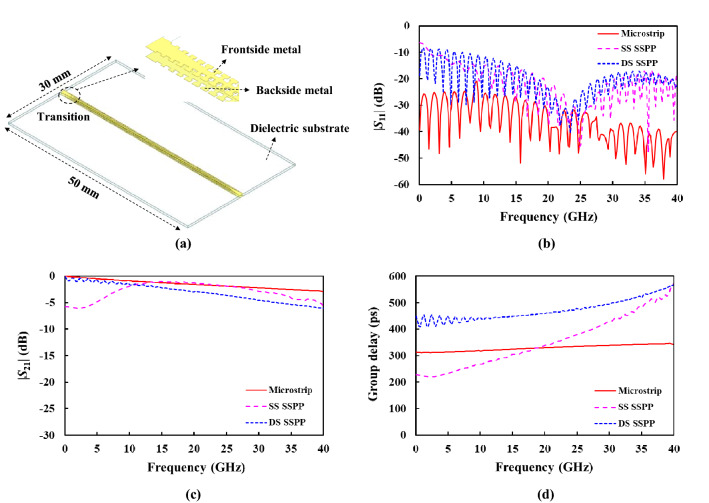
Single straight transmission line. (**a**) DS SSPP line. (**b**) Simulated $| {S_{11} } |$. (**c**) Simulated $| {S_{21} } |$. (**d**) Simulated group velocity.

A single straight transmission line can be implemented by repeatedly interconnecting the unit-cell structures of Fig. 1, as illustrated in Fig. 3a. Note that the transition structures are inserted between the DS SSPP line and the ports, for the simulation purpose. Its design will be discussed later. The wave ports of HFSS are used for this simulation. The SS SSPP shows a frequency-dependent port impedance of 177.1 ± 30.3 Ω from 0.1 to 40.0 GHz, so that it is not suited for the broadband signal transmission because of its high and variable impedance. It requires a complex transition structure to the conventional 50-Ω system. On the contrary, the DS SSPP and the microstrip lines exhibit almost constant port impedances of 59.0 ± 1.0 Ω and 46.5 ± 1.5 Ω, respectively, over the same frequency range. The width of the microstrip line is 1.0 mm which is the same as the outer width ($W$) of the DS SSPP line. Figure 3b,c shows the simulated $| {S_{11} } |$ and $| {S_{21} } |$ of the transmission lines, respectively. The microstrip line shows the best impedance matching performance or return loss ($- 20\log | {S_{11} } |$). The SS SSPP line exhibits a large fluctuation in the insertion loss ($- 20\log | {S_{21} } |$) with frequency, the highest loss from 0.1 to 10.0 GHz and the lowest loss from 15.0 to 30.0 GHz among three transmission lines. On the contrast, the DS SSPP line exhibits the monotonically increasing loss with frequency like the microstrip line, but with a higher loss than the microstrip line. The group delay of 50.0 mm-long transmission lines is also calculated from the simulated *S*-parameters. Figure 3d demonstrates that the DS SSPP exhibits a relatively constant group delay with frequency, compared with the SS SSPP line, as expected from the simulated group velocities of the unit cells.

In summary, the DS SSPP line is well-suited for the broadband signal transmissions, because it allows the SSPP mode nearly up to cut-off frequency with small variation in the characteristic impedance and group velocity (or group delay).

**Figure 4:**
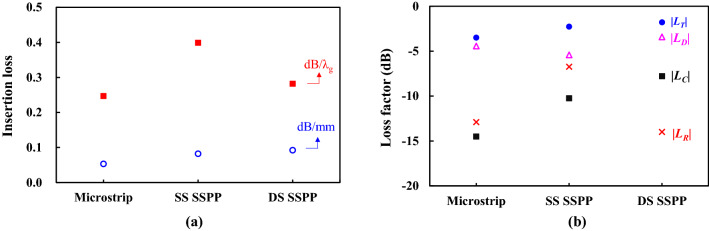
Loss analysis of straight transmission lines at 35.0 GHz. (**a**) Calculated insertion loss per unit length and per a wavelength. (**b**) Loss components.

Figure 4 shows the loss analysis of the straight transmission lines. In Fig. 4a, the insertion loss per unit length (mm) and per a wavelength ($\lambda_{g}$) are compared at 35.0 GHz which is below the intersection frequency of the SS SSPP and the microstrip lines, and thus the SS SSPP line has a longer wavelength than the microstrip line. The DS SSPP line shows the highest loss per mm (0.092 dB/mm) at 35.0 GHz. The difference of the loss per a wavelength between the microstrip line and DS SSPP line becomes smaller, because the DS SSPP has shorter wavelength (3.06 mm at 35.0 GHz) which is only 66.4% of that of the microstrip line.

Another loss analysis is carried out to find the loss component associated with each line. Total loss ($| {L_{T} } |$) produced inside the transmission line consists of conductor loss ($| {L_{C} } |$), dielectric loss ($| {L_{D} } |$), and radiation loss ($| {L_{R} } |$). That is,

2 $$ | {L_{T} } |^{2} = | {L_{C} } |^{2} + | {L_{D} } |^{2} + | {L_{R} } |^{2} = { }1 - \mathop \sum \limits_{i = 1}^{2} | {S_{i1} } |^{2} . $$

The conductor loss can be extracted by comparing the total losses of two simulations, one using the conductors with real conductivity and the other using conductors as PECs. The dielectric loss can be also extracted in the similar way (one with real $\tan \delta$ and the other with $\tan \delta = 0$. The radiation loss is finally calculated from Eq. (2) using the extracted conductor and dielectric losses. Figure 4b shows the loss components calculated at 35.0 GHz. The dominant loss is caused by the dielectric in all the three transmission lines. The DS SSPP line has the relatively high dielectric and conductor losses, because of the tight field confinement around metal strips. For the same reason, it shows the smallest radiation loss among the three transmission lines. This advantage can be more distinct in the coupled or curved lines, as will be shown in the next section about the coupled lines and ring resonators. The SS SSPP line exhibits a higher radiation loss than even the microstrip line because the surface-wave property diminishes at the frequency lower than the intersection frequency.

**Figure 5:**
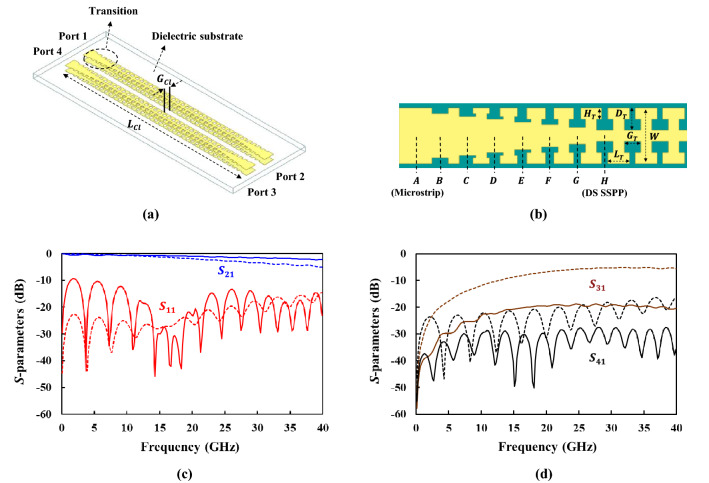
Coupled transmission lines and simulated *S*-parameters in microstrip (slotted) and DS SSPP (solid). (**a**) DS SSPP coupled lines. (**b**) Microstrip-to-DS SSPP transition in which $H_{T}$ = 0.20 mm, $D_{T}$ = 0.40 mm, $G_{T}$ = 0.30 mm, $L_{T}$ = 0.40 mm, and $W$ = 1.00 mm. (**c**) Simulated insertion and return losses. (**d**) Simulated coupling ($| {S_{31} } |$) and isolation ($| {S_{41} } |$).

The designed DS SSPP exhibits a strong field confinement around metal strips, so that it is helpful in reducing the cross-talks or coupling between closely placed transmission lines. In order to prove this fact, the DS SSPP coupled lines with microstrip-to-DS SSPP transition was designed as shown in Fig. 5a,b. The comparison of the simulated performance between the DS SSPP and microstrip coupled lines are presented in Fig. 5c,d, where the gap distance between two lines ($G_{cl}$) is 1.0 mm and the line length ($L_{cl}$) is 20.0 mm (~ 6.5 $\lambda_{g}$ at 35.0 GHz). The lumped ports can be closely placed and are used for the simulations of the microstrip and DS SSPP coupled lines. However, the single-conductor SS SSPP line can be simulated using the wave ports. Two wave ports in the SS SSPP coupled lines overlap with each other, so that the SS SSPP coupled lines are excluded in the comparison. Unlike the single straight line in the previous section, the DS SSPP coupled lines show a lower insertion loss to through port (port 2) than the microstrip line by 1.5 dB on average from 10.0 to 40.0 GHz as shown Fig. 5c. This is because the former has a lower coupling to port 3 ($| {S_{31} } |$) than the microstrip line by 14.2 dB on average over entire frequency range as shown Fig. 5d. The DS SSPP coupled lines also show a better isolation ($| {S_{41} } |$) (> 30.0 dB) higher than the microstrip line for the same reason. These results are caused by the tight field confinement of the designed DS SSPP line which reduces the coupling to adjacent lines and achieve high isolation. Therefore, the DS SSPP line can accomplish lower-loss transmission to the through port with the reduced cross-talks and interferences compared to the microstrip line, for the case where many lines are closely placed within a compact size. It can be also inferred from the dispersion curves in Fig. 2a that the DS SSPP lines can exhibit the reduced coupling than the SS SSPP lines.

**Figure 6:**
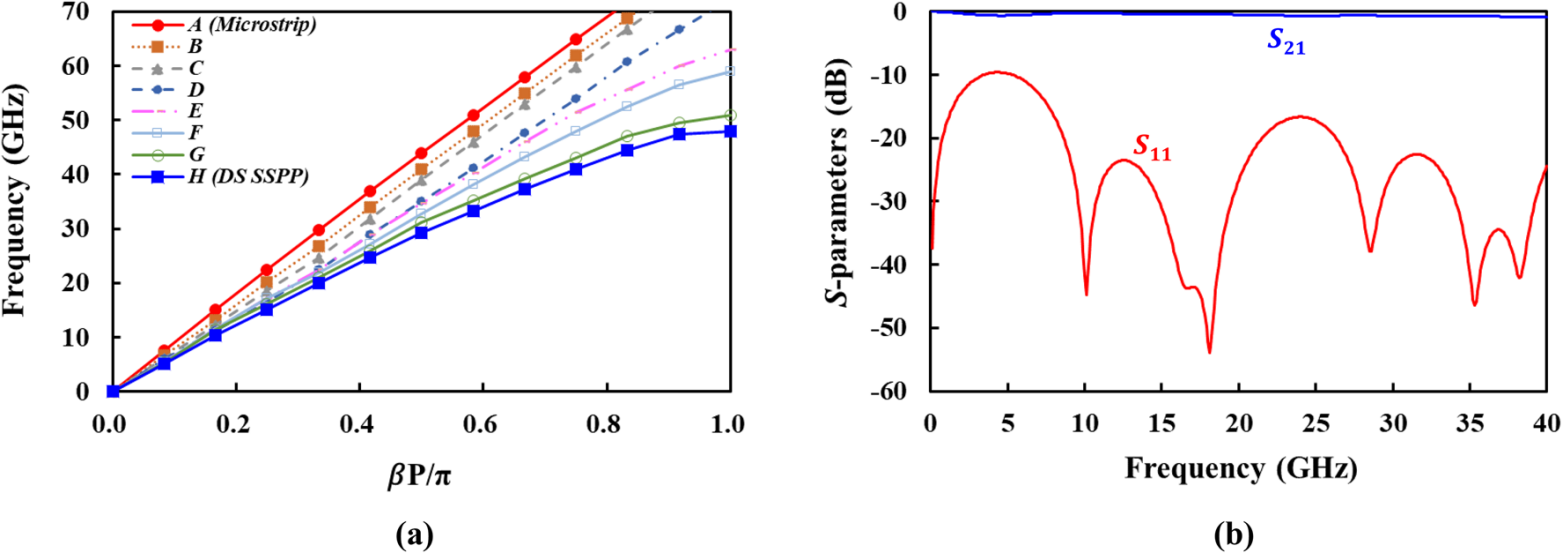
Design of microstrip-to-DS SSPP transition. (**a**) Dispersion curves at different positions along transition. (**b**) Simulated *S*-parameters of back-to-back transition.

For the measurement of the DS SSPP lines, the transition structure was designed to efficiently excite SSPP mode from the microstrip lines and to achieve the momentum and impedance matching between the microstrip and DS SSPP lines. The short transition can cause the mismatches at low frequencies, resulting in the ripples and stopband in the insertion loss^9^. Figure 5b shows the designed microstrip-to-DS SSPP transition using gradient corrugation grooves, where the groove depth gradually increases from the position *A* (microstrip) and forms *T*-shaped SSPP at the position *H* (DS SSPP). As shown in Fig. 6a, the dispersion curve of the microstrip line at *A* gets close to that of the DS SSPP line at *H* along the transition. The simulated performance of the back-to-back transition is given in Fig. 6b, showing an insertion loss less than 0.9 dB and return loss better than 9.6 dB, respectively, from 0.1 to 40.0 GHz. The coaxial-to-microstrip transition, where coaxial adaptor is connected to the microstrip line through the grounded coplanar waveguide is also designed and optimized.

### Measurement results of DS SSPP lines

**Figure 7:**
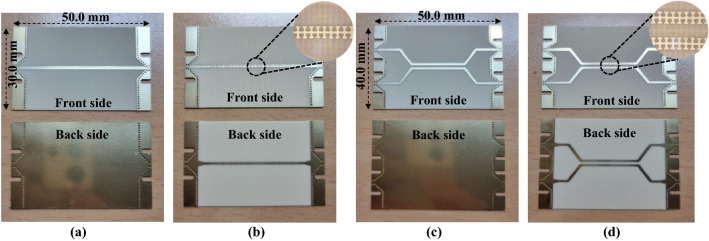
Fabricated transmission lines. (**a**) Microstrip straight line. (**b**) DS SSPP straight line. (**c**) Microstrip coupled lines. (**d**) DS SSPP coupled lines.

The designed single-straight and coupled lines in the DS SSPP and microstrip were fabricated as shown in Fig. 7, where end-launch coaxial adaptors are omitted. For the coupled lines, two lines are flared to accommodate two adaptors on the same side. The performance was measured by the two-port vector network analyzer after short-open-load-through calibrations.

**Figure 8:**
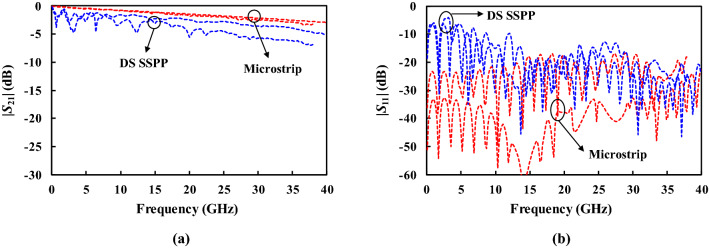
Measured (solid) and simulated (slotted) results of microstrip and DS SSPP lines. (**a**) $| {S_{21} } |$. (**b**) $| {S_{11} } |$.

Figure 8 shows the measured *S*-parameters of 50 mm-long straight lines in the DS SSPP and microstrip. Similar to the simulation results, the DS SSPP line exhibits relatively higher insertion loss than the microstrip line as shown in Fig. 8a, due to the dielectric and conductor losses. It shows more pronounced low-frequency ripples in the insertion loss than the simulation, which seems to be caused by the fabrication errors in the coaxial-to-microstrip-to-DS SSPP transition^9^.

**Figure 9:**
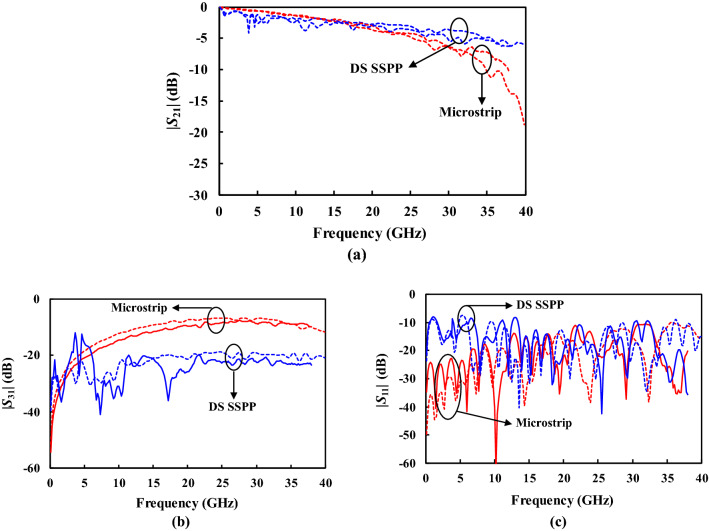
Measured (solid) and simulated (slotted) results of microstrip and DS SSPP coupled lines for $G_{cl}$ = 1.00 mm. (**a**) $| {S_{21} } |$. (**b**) $| {S_{31} } |$. (**c**) $| {S_{11} } |$.

Figure 9 shows the measured *S*-parameters of the coupled lines with a gap distance of 1.0 mm. The unmeasured ports in the coupled lines were terminated with 50 Ω-loads. The designed DS SSPP coupled lines present the lower insertion loss ($| {S_{21} } |$) by 1.4 dB on average from 20.0 to 38.0 GHz, and the lower coupling ($| {S_{31} } |$) by 13.8 dB on overage from 6.5 to 38.0 GHz, compared to the microstrip coupled line. From this result, it can be stated that the DS SSPP lines enforce the EM fields to be confined around the metal strips so that the coupling to adjacent lines can be greatly reduced. It leads to the lower insertion loss to the through port. It is also beneficial for reducing the cross-talks between two closely spaced lines allowing high-quality signal transmissions. The measured return losses have good agreement with the simulation for both the microstrip and the DS SSPP lines as shown in Fig. 9c.

**Figure 10:**
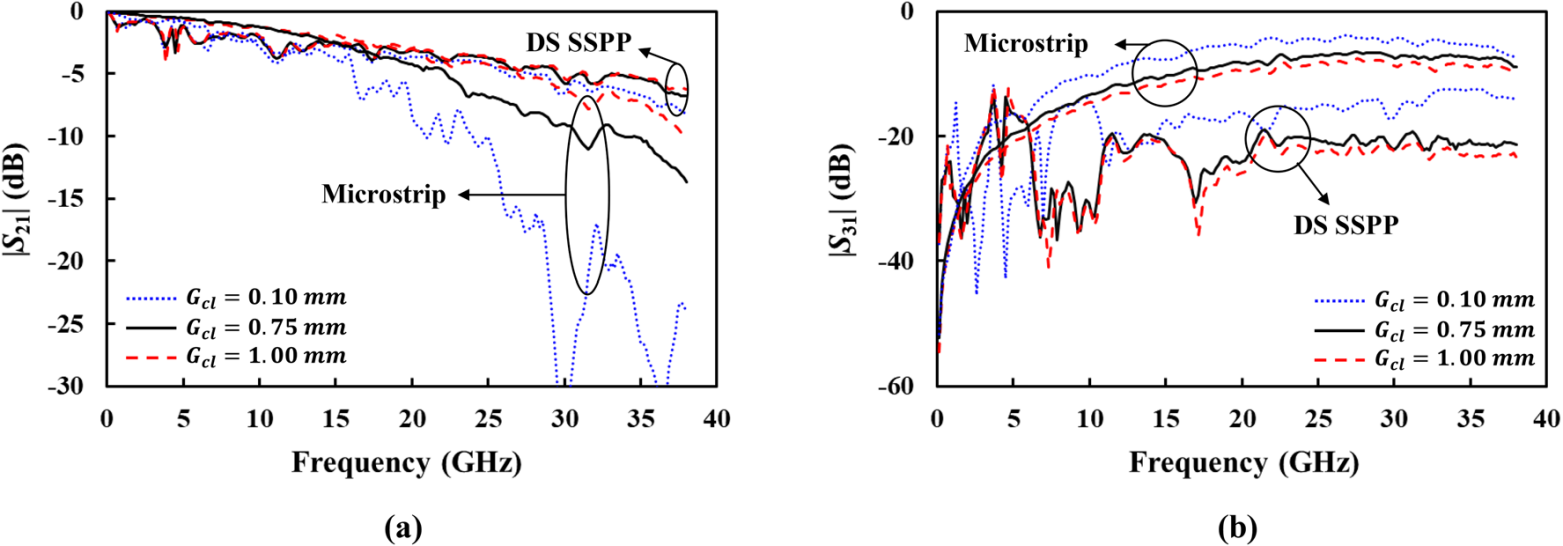
Measured results of microstrip and DS SSPP coupled lines for $G_{cl}$ = 0.10 (dotted), 0.75 (solid), and 1.00 mm (slotted). (**a**) $| {S_{21} } |$. (**b**) $| {S_{31} } |$.

The DS SSPP lines allow the reduced coupling at the gap distances smaller than 1.00 mm as well. Figure 10 shows the measured *S*-parameters of the coupled lines for various gap distances ($G_{cl}$ = 0.10, 0.75, and 1.00 mm). As expected, as two lines get closer, or $G_{cl}$ reduces, the coupling increases and thus the insertion loss to the through port also increases for both the microstrip and DS SSPP lines as shown in Fig. 10a,b. The designed DS SSPP exhibits better performance such as lower coupling from 7.0 to 38.0 GHz and lower insertion loss from 20.0 to 38.0 GHz. Especially, it should be noted that the DS SSPP coupled lines with $G_{cl}$ = 0.10 mm have the lower coupling and the lower insertion loss than the microstrip line with $G_{cl}$ = 1.00 mm at the frequency > 30.2 GHz. Therefore, the designed DS SSPP lines can be more closely placed in a given area with the reduced insertion loss and cross-talks, allowing the dense interconnects and compact circuit area.

### Application to microwave resonators

The SSPP line exhibits the tight field confinement leading to the reduction of the EM leakage and the radiation loss. It also has a short wavelength allowing compact microwave circuits. Various microwave circuits in the SSPP have been reported, such as power dividers/combiners^10,11^, frequency splitters^11,12^, resonators^13^, filters^14,15^, and antennas^16,17^. Based on the spoof localized surface plasmons (SLSPs), the plasmonic structures using subwavelength corrugations have been proposed for the applications to the sensors and bandpass filters^18,19^. The SLSP-based toroidal plasmonic resonators consisting of double-layer spoke patterns have been designed to have a high *Q*-factor with the reduced circuit size^20^. In this work, the microwave ring resonator using the designed *T*-shaped DS SSPP is presented, providing higher $Q$-factor than the microstrip resonator with a smaller chip size.

**Figure 11:**
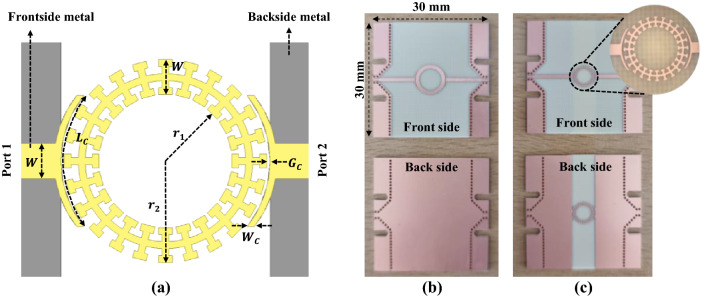
Microwave ring resonators. (**a**) Layout of DS SSPP ring resonator. (**b**) Fabricated microstrip ring resonator. (**c**) Fabricated DS SSPP ring resonator. Both resonators have the same coupling structure in which $$ W_{c}$$ = 0.20 mm, $$L_{c}$$ = 4.05 mm, and $$G_{c}$$ = 0.10 mm.

Figure 11a shows the layout of the designed ring resonator in the *T*-shaped DS SSPP. The fabricated microstrip and DS SSPP ring resonators are shown in Fig. 11b,c, respectively. At resonant frequency ($f_{r}$), the following relation is satisfied

3 $$ 2\pi R = m\lambda_{g} ( {m = 1,\;2,\;3 \ldots } ), $$

where $r_{1}$ and $r_{2}$ are inner and outer radii of the ring, respectively, and $R = ( { r_{1} + r_{2} } )/2$. In this work, $r_{1}$ and $r_{2}$ are determined to be 1.90 and 2.90 mm, respectively, for $f_{r}$ = 7.5 GHz. For the microstrip line, $r_{1}$ = 2.85 mm and $r_{2}$ = 3.85 mm allow the same resonant frequency. Therefore, the DS SSPP ring resonator occupies only 56.8% of the chip area of the microstrip ring resonator. The same gap-coupled structure with microstrip feed lines is used for both DS SSPP and microstrip ring resonators as shown in Fig. 11.

**Figure 12:**
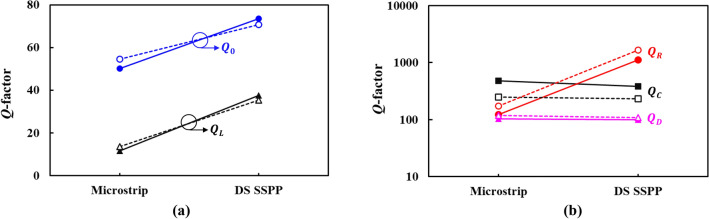
Calculated $Q$-factors of ring resonators at fundamental (slotted) and second harmonic (solid) resonant frequencies. (**a**) $Q_{L}$ and $Q_{0}$. (**b**) $Q_{C}$, $Q_{D}$, and $Q_{R}$.

In order to compare the performance of the DS SSPP and microstrip resonators, the loaded and unloaded *Q*-factors ($Q_{L}$ and $Q_{0}$, respectively) are extracted from the simulated *S*-parameters^21^ and compared in Fig. 12a. It is shown that the DS SSPP resonator has higher $Q_{0}$ than the microstrip resonator at both the fundamental and 2nd harmonic resonant frequencies. This is caused by the tight field confinement and the reduced radiation loss in the DS SSPP ring resonator. This can be proved by calculating the $Q$-factors individually, or $Q_{C}$, $Q_{D}$, and $Q_{R}$ which are *Q*-factors contributed by the conductor, dielectric, and radiation losses, respectively. The $Q_{0}$ is given by

4 $$ \frac{1}{{Q_{0} }} = \frac{1}{{Q_{C} }} + \frac{1}{{Q_{D} }} + \frac{1}{{Q_{R} }}.{ } $$

Figure 12b shows the calculated $Q_{C}$, $Q_{D}$, and $Q_{R}$ for the DS SSPP and microstrip ring resonators. It can be stated that the DS SSPP has higher $Q_{0}$ than the microstrip line thanks to the dramatic reduction of the radiation loss, which is noticeable in this curved transmission lines. Even though the DS SSPP needs the smaller radius of the ring and suffers from larger curvature, it shows the lower EM wave leakage due to the strong surface wave property than the microstrip line. It is also worthwhile to state that the DS SSPP resonator has higher $Q_{0}$ at $2f_{r}$ than that at $f_{r}$, which is opposite to the conventional microstrip resonator, as shown in Fig. 12a. As the frequency increases, the conductor *Q*-factor $Q_{C}$ improves for both the DS SSPP and microstrip resonators as shown in Fig. 12b^22^. However, this effect on the $Q_{0}$ of the microstrip resonator is weakened by the high radiation loss. On the contrary, it effectively increases the $Q_{0}$ of the DS SSPP ring resonators due to the reduced radiation loss.

**Figure 13:**
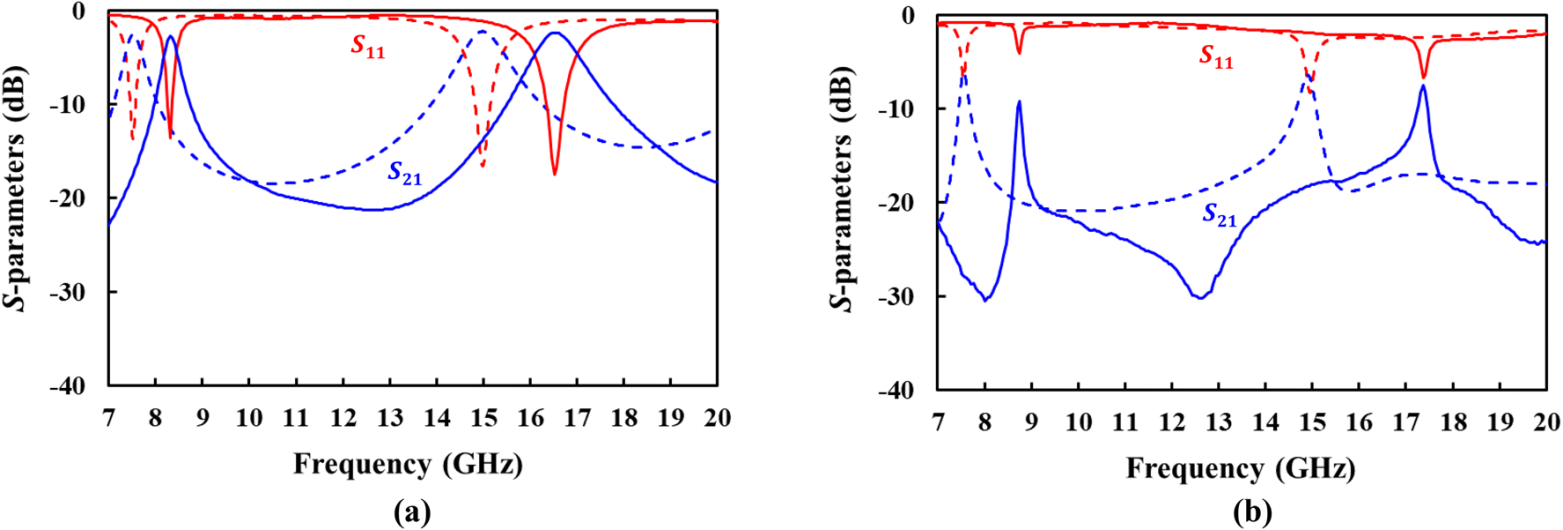
Measured (solid) and simulated (slotted) *S*-parameters of (**a**) microstrip and (**b**) DS SSPP ring resonators.

Figure 13 shows the measured *S*-parameters of fabricated ring resonators. The resonant frequencies shift upward compared to the simulation, which is caused by the pattern fabrication errors. The dimensions of the fabricated metal strips were measured by using the contact profiler and it was found that the metal strips were over-etched by ~ 150 μm. Anyway, the DS SSPP resonator shows the high $Q_{0}$ of 107.9 and 135.3 at fundamental and 2nd harmonic resonant frequencies, respectively, as listed in Table 1, which corresponds to 1.3- and 2.2-times improvement compared to the microstrip ring resonator. Therefore, the DS SSPP ring resonators provide high *Q*-factors as a result of the reduced radiation loss under small circuit size. It is also worthwhile to note that as shown in Fig. 13, the microstrip ring resonator exhibits the stronger coupling (or higher |*S*_21_| at the resonant frequency) than the DS SSPP ring resonator, because it operates in the same quasi-TEM mode as the microstrip feed lines.

**Table 1:** Measured resonant frequencies ($f_{r}$’s) and unloaded $Q$-factors ($Q_{0}$’s) of ring resonators.

Resonator	$f_{r}$ (GHz)	$Q_{0}$ at $f_{r}$	$Q_{0}$ at $2f_{r}$
Microstrip	8.3	81.9	62.8
DS SSPP	8.7	107.9	135.3

## Discussion

In this work, the *T*-shaped DS SSPP line operating up to 40 GHz was proposed, which present the strong surface-wave property from very low to very high frequencies. It is shown that it has more constant group velocity according to the frequency without the intersection frequency, compared to the counterpart SS SSPP line. In addition, this two-conductor transmission line allows easy interconnection with the active devices and TEM-based transmission lines using a compact transition structure.

Compared to the microstrip lines, the designed DS SSPP lines showed the lower coupling between two adjacent lines and the lower insertion loss to through port, for various line spacings. Therefore, they can be more densely packed in the given area with less cross-talks or interferences. The designed DS SSPP showed the strong field confinement leading to the reduction of the radiation loss. This property was applied to design the microwave ring resonators which showed the higher *Q*-factors at both the fundamental and 2nd harmonic frequencies, compared to the microstrip ring resonators. The reduced wavelength of the DS SSPP lines also allowed the miniaturization of microwave circuits.

In summary, the designed DS SSPP can be effectively applied for the high-speed interconnects with signal-integrity under a compact area. It can also be applied for the design of the high-performance miniaturized microwave and millimeter-wave circuits.

## Methods

Numerical simulations are performed by using the commercial software, HFSS by Ansys, Inc. The dispersion curves are obtained by calculating the eigen frequencies of the unit cell structure with different phase shifts between the master and slave boundaries in y-direction using eigen-mode solution. A high-TG FR-4 substrate is used for the fabrication of the lines and resonators. The vector network analyzer (by Agilent Technologies, Inc.) is used to measure the $S$-parameters. The loaded *Q*-factor of the resonator is extracted from |$S_{21}$| by using $Q_{L} = f_{r} /BW$, where $BW$ is a half-power bandwidth^21^. The unloaded *Q*-factor is then computed by using $Q_{0} = ( {1 + g} )Q_{L}$, where $g$ is a coupling coefficient given by $g = S_{21} ( {f_{r} } )/( {1 - S_{21} ( {f_{r} } )} )$.
